# Optimized Hyper Beamforming of Linear Antenna Arrays Using Collective Animal Behaviour

**DOI:** 10.1155/2013/982017

**Published:** 2013-07-22

**Authors:** Gopi Ram, Durbadal Mandal, Rajib Kar, Sakti Prasad Ghoshal

**Affiliations:** ^1^Department of Electronics and Communication Engineering, National Institute of Technology, Durgapur, India; ^2^Department of Electrical Engineering, National Institute of Technology, Durgapur, India

## Abstract

A novel optimization technique which is developed on mimicking the collective animal behaviour (CAB) is applied for the optimal design of hyper beamforming of linear antenna arrays. Hyper beamforming is based on sum and difference beam patterns of the array, each raised to the power of a hyperbeam exponent parameter. The optimized hyperbeam is achieved by optimization of current excitation weights and uniform interelement spacing. As compared to conventional hyper beamforming of linear antenna array, real coded genetic algorithm (RGA), particle swarm optimization (PSO), and differential evolution (DE) applied to the hyper beam of the same array can achieve reduction in sidelobe level (SLL) and same or less first null beam width (FNBW), keeping the same value of hyperbeam exponent. Again, further reductions of sidelobe level (SLL) and first null beam width (FNBW) have been achieved by the proposed collective animal behaviour (CAB) algorithm. CAB finds near global optimal solution unlike RGA, PSO, and DE in the present problem. The above comparative optimization is illustrated through 10-, 14-, and 20-element linear antenna arrays to establish the optimization efficacy of CAB.

## 1. Introduction

Beamforming is a signal processing technique used to control the directionality of the transmission and reception of the radio signals [1]. This is achieved by distributing the elements of the array in such a way that signals at particular angles experience constructive interference while others experience destructive interference. Beamforming can be used at both transmitting and receiving ends in order to achieve spatial selectivity. Hyper beamforming refers to spatial processing algorithm used to focus an array of spatially distributed elements (called sensors) to increase the signal to interference plus noise ratio at the receiver. This beamforming processing improves significantly the gain of the wireless link over a conventional technology, thereby increasing range, rate, and penetration [2–4]. It has found numerous applications in radar, sonar, seismology, wireless communication, radio astronomy, acoustics, and biomedicine [5]. It is generally classified as either conventional (switched and fixed) beamforming or adaptive beamforming. Switched beamforming system [6, 7] is a system that can choose one pattern from many predefined patterns in order to enhance the received signals. Fixed beamforming uses fixed set of weights and time delays (or phasing) to combine the signals received from the sensors in the array, primarily using only information about the locations of the sensors in space and the wave direction of interest [8]. Adaptive beamforming is based on the desired signal maximization mode and interference signal minimization mode [9–11]. It is able to place the desired signal at the maximum of main lobe. The hyper beamforming/any other beamforming offers high detection performance like beam width, the target bearing estimation and reduces false alarm, sidelobe suppression. A new optimized hyper beamforming technique is presented in this paper, and collective animal behaviour (CAB) approach is applied to obtain optimal hyperbeam patterns [12, 13] of linear antenna arrays.

The classical gradient-based optimization methods are not suitable for optimal design of hyper beamforming of linear antenna arrays due to the following reasons: (i) “highly sensitive to starting points when the number of solution variables and hence the size of the solution space increase,” (ii) frequent convergence to local optimum solution or divergence or revisiting the same suboptimal solution, (iii) requirement of continuous and differentiable objective function (with gradient search methods), (iv) requirement of the piecewise linear cost approximation (linear programming), and (v) problem of convergence and algorithm complexity (with nonlinear programming). So, evolutionary methods have been employed for the optimal design of hyper beamforming of linear antenna arrays with better parameter control. Different evolutionary optimization algorithms such as simulated annealing algorithms [14] and genetic algorithm (GA) [15–19] have been widely used to the synthesis of design methods capable of satisfying constraints which would be unattainable. When considering global optimization methods for antenna arrays design, GA seems to be the promising one. Standard GA (herein referred to as real coded GA (RGA)) has a good performance for finding the promising regions of the search space, but it is prone to revisiting the same suboptimal solutions.

Particle swarm optimization (PSO) is an evolutionary algorithm developed by Kennedy and Eberhart [20]. PSO is simple to implement, and its convergence may be controlled via few parameters. The limitations of the conventional PSO are that it may be influenced by premature convergence and stagnation problem [21–30].

DE algorithm [31–42] was first introduced by Storn and Price in 1995 [31]. Like RGA, it is a randomized stochastic search technique enriched with the operations of crossover, mutation, and selection. DE is also prone to premature convergence and stagnation. So, to enhance the performance of optimization algorithms in global search (exploration stage) as well as local search (exploitation stage), the authors suggest an alternative technique as collective animal behaviour (CAB) algorithm for the optimization problem of hyper beamforming.

The rest of the paper is arranged as follows. In Section 2, the design equations of hyper beamforming of linear antenna array are formulated.  Section 3 briefly discusses on evolutionary algorithms RGA, PSO, DE, and CAB employed for the designs.  Section 4 describes the simulation results obtained by employing the algorithms. Finally, Section 5 concludes the paper.

## 2. Design Equations

**Figure 1:**
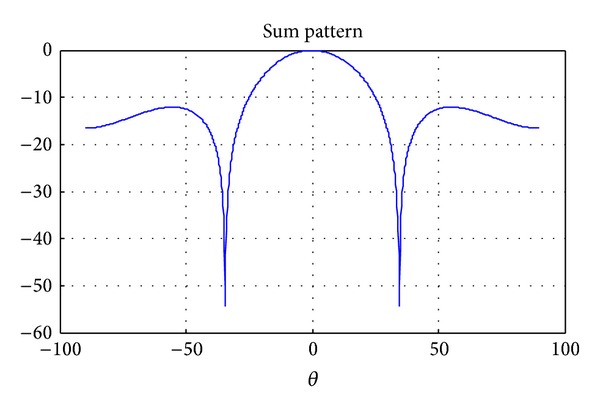
Sum beam pattern for the 10-element linear array for *u* = 1.

**Figure 2:**
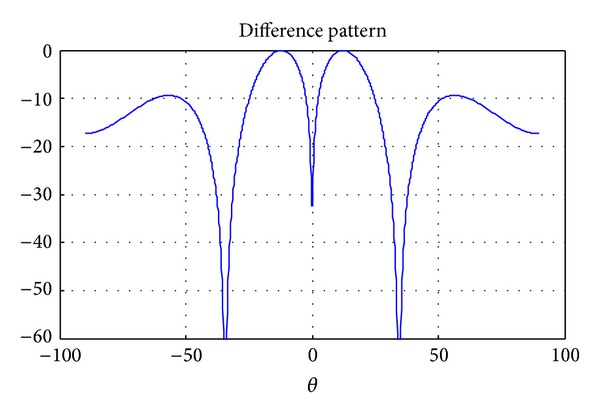
Difference beam pattern for the 10-element linear array for *u* = 1.

Hyperbeam technique generates a narrow beam as compared to conventional beam with improved performance of SLL and FNBW that depend on the variation of exponent parameter value (*u*). In hyper beamforming for linear antenna array, the interelement spacing in either direction is *λ*/2 in order to steer the beam in that particular direction. The sum beam can be created by summation of the absolute values of complex left and right half beams, as shown in Figure 1. The difference beam is the absolute magnitude of the difference of complex right beam half beam and left half beam signals. Furthermore, the difference beam has a minimum in the direction of the sum beam at zero degree as shown in Figure 2. The resulting hyperbeam is obtained by subtraction of sum and difference beams, each raised to the power of the exponent *u*.

**Figure 3:**
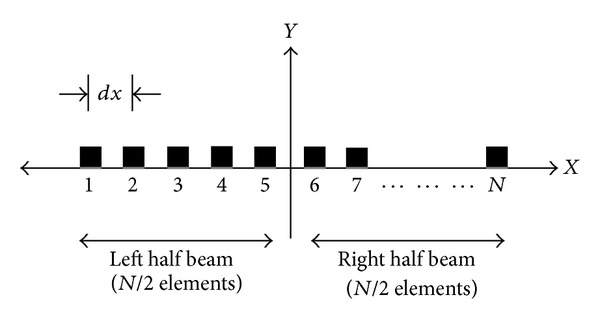
Geometry of an *N*-element linear array along the *x*-axis.

Consider a broadside linear array of *N* equally spaced isotropic elements as shown in Figure 3. The array is symmetric in both geometry and excitation with respect to the array center [8].

For broadside beams, the array factor is given in [6]:

(1) $$\begin{array}{c} \text{AF}\left(\theta \right)=\overset{N}{\underset{n=1}{\sum }}I_{n}\;e^{j(n-1)Kd[\sin\theta \cos\phi -\sin\theta_{0}\cos\phi_{0}]}, \end{array}$$

where *θ* = angle of radiation of electromagnetic plane wave; *d* = interelement spacing; *K* = propagation constant; *N* = total number of elements in the array; *I*_*n*_ = excitation amplitude of *n*th element.

The equations for the creation of sum, difference, and simple hyperbeam pattern in terms of two half beams are as follows [8]:

 
*Sum Pattern*
  (2) $$\begin{array}{c} \;\;\text{Sum}\left(\theta \right)=\left|R_{L}\right|+\left|R_{R}\right|, \end{array}$$
 
*Difference Pattern*
  (3) $$\begin{array}{c} \text{Diff}\left(\theta \right)=\left|R_{L}-R_{R}\right|, \end{array}$$

where

(4) $$\begin{array}{c} R_{L}=\overset{N/2}{\underset{n=1}{\sum }}I_{n}\;e^{j(n-1)Kd[\sin\theta \cos\phi -\sin\theta_{0}\cos\phi_{0}]},\\ R_{R}=\overset{N}{\underset{n=N/2+1}{\sum }}I_{n}\;e^{j(n-1)Kd[\sin\theta \cos\phi -\sin\theta_{0}\sin\phi_{0}]}. \end{array}$$

Hyperbeam is obtained by subtraction of sum and difference beams, each raised to the power of the exponent *u*; the general equation of hyperbeam is a function of hyperbeam exponent *u* as given in

(5) $$\begin{array}{c} \text{A}{\text{F}}_{\text{Hyper}}\left(\theta \right)={\left\{{\left(\left|R_{L}\right|+\left|R_{R}\right|\right)}^{u}-{\left(\left|R_{L}-R_{R}\right|\right)}^{u}\right\}}^{1/u}, \end{array}$$

where *u* ranges from 0.2 to 1. If *u* lies below 0.2, hyperbeam pattern will contain a large depth spike at the peak of the main beam without changing in the hyperbeam pattern. If *u* increases more than 1, sidelobes of hyperbeam will be more as compared to conventional radiation pattern.

All the antenna elements are assumed isotropic. Only amplitude excitations and interelement spacing are used to change the antenna radiation pattern. The cost function (CF) for improving the SLL of radiation pattern of hyperbeam linear antenna arrays is given in

(6) $$\begin{array}{c} \text{CF}=\mathrm{Max}\frac{\left|\text{A}{\text{F}}_{\text{Hyper}}\left(\theta_{\text{msl}1},I_{n}\right)\right|}{\left|\text{A}{\text{F}}_{\text{Hyper}}\left(\theta_{0},I_{n}\right)\right|}+\mathrm{Max}\frac{\left|\text{A}{\text{F}}_{\text{Hyper}}\left(\theta_{\text{msl}2},I_{n}\right)\right|}{\left|\text{A}{\text{F}}_{\text{Hyper}}\left(\theta_{0},I_{n}\right)\right|}, \end{array}$$

where *θ*_0_ is the angle where the highest maximum of central angle is attained in *θ* ∈ [−*π*/2, *π*/2]. *θ*_msl1_ is the angle where maximum side lobe AF_Hyper_(*θ*_msl1_, *I*_*n*_) is attained in the lower band of hyperbeam pattern. *θ*_msl2_ is the angle where the maximum sidelobe AF_Hyper_(*θ*_msl2_, *I*_*n*_) is attained in the upper side band of hyperbeam pattern. In CF, both numerator and denominator are in absolute magnitude. Minimization of CF means maximum reduction of SLL. RGA, PSO, DE, and CAB are employed individually for minimization of CF by optimizing current excitation weights of elements and interelement spacing. Results of the minimization of CF and SLL are described in Section 4.

## 3. Optimization Technique Employed

### 3.1. Real Coded Genetic Algorithm (RGA)

Real coded genetic algorithm (RGA) is mainly a probabilistic search technique, based on the principles of natural selection and evolution. At each generation, it maintains a population of individuals where each individual is a coded form of a possible solution of the problem at hand called chromosome. Chromosomes are constructed over some particular alphabet, for example, the binary alphabet {0,1}, so that chromosomes' values are uniquely mapped onto the real decision variable domain. Each chromosome is evaluated by a function known as fitness function, which is usually the objective function of the corresponding optimization problem [15–19].

The basic steps of RGA are shown as follows.

*Step  1*. Initialize the real chromosome strings of *n*_*p*_ population, each consisting of a set of coefficients of current excitation weights and interelement spacing CF.

*Step  2*. Decoding the strings and evaluation of each string.

*Step  3*. Selection of elite strings in order to increase CF values from the minimum value.

*Step  4*. Copying the elite strings over the nonselected strings.

*Step  5*. Crossover and mutation generate the offsprings.

*Step  6*. Genetic cycle updating.

*Step  7*. The iteration stops when the maximum number of cycles is reached. The grand minimum CF and its corresponding chromosome string or the desired solution of coefficients of optimal current excitation weights and optimal interelement spacing are finally obtained.

### 3.2. Particle Swarm Optimization (PSO)

PSO is a flexible, robust population-based stochastic search or optimization technique with implicit parallelism, which can easily handle with nondifferential objective functions, unlike traditional gradient-based optimization methods. PSO is less susceptible to getting trapped on local optima unlike GA, simulated annealing, and so forth. Eberhart et al. developed PSO concept similar to the behaviour of a swarm of birds [20–30]. PSO is developed through simulation of bird flocking and fish schooling in multidimensional space. Bird flocking optimizes a certain objective function. Each particle knows its best value so far (*pbest*). This information corresponds to personal experiences of each particle. Moreover, each particle knows the best value so far in the group (*gbest*) among all *pbest*s. Namely, each particle tries to modify its position using the following information:

- the distance between the current position and the *pbest*;
- the distance between the current position and the *gbest*.

Mathematically, velocities of the vectors are modified according to the following equation:

(7) Vik+1=CFa×(wk+1∗Vik+C1∗rand1∗(pbesti−Sik)    + C2∗rand2∗(gbestk−Sik)),

where *V*_*i*_^*k*^ is the velocity of vector *i* at iteration *k*; *w* is the weighting function; *C*_1_ and *C*_2_ are called social and cognitive constants, respectively; rand_*i*_ is the random number between 0 and 1; *S*_*i*_^*k*^ is the current position of vector *i* at iteration *k*; *pbest*_*i*_ is the *pbest* of vector *i*; *gbest* ^*k*^ is the *gbest* of the group of vectors at iteration *k*. The first term of (7) is the previous velocity of the vector. The second and third terms are used to change the velocity of the vector. Without the second and third terms, the vector will keep on “flying” in the same direction until it hits the boundary. The parameter *w* corresponds to a kind of inertia and tries to explore new areas. Here, the vector is termed for the string of real current excitation weight coefficients (*N* number) and uniform interelement spacing (01 number). Total number of variables = *n*_var_ = *N* + 1 in each vector.

Normally, *C*_1_ = *C*_2_ = 1.5–2.05, and constriction factor (CFa) is given in

(8) $$\begin{array}{c} \text{CFa}=\frac{2}{\left|2-\varphi -\sqrt{\varphi^{2}-4\varphi }\right|}, \end{array}$$

where

(9) $$\begin{array}{c} \varphi =C_{1}+C_{2},\;\varphi >4. \end{array}$$

For *C*_1_ = *C*_2_ = 2.05, the computed value of CFa = 0.73.

The best values of *C*_1_, *C*_2_, and CFa are found to vary with the design sets.

Inertia weight (*w* ^*k*+1^) at (*k* + 1)th cycle is as given in

(10) $$\begin{array}{c} w^{k+1}=w_{\max}-\frac{w_{\max}-w_{\min}}{k_{\max}}\times \left(k+1\right), \end{array}$$

where *w*_max⁡_ = 1.0; *w*_min⁡_ = 0.4; *k*_max⁡_ = maximum number of iteration cycles. The searching point/updated vector in the solution space can be modified by

(11) $$\begin{array}{c} S_{i}^{k+1}=S_{i}^{k}+V_{i}^{k+1}. \end{array}$$

The basic steps of PSO are shown as follows. 

*Step  1 (initialization)*. Population (swarm size) of particle vectors, *n*_*p*_ = 120; maximum iteration cycles = 100; *N* number of current excitation weights and one number uniform interelement spacing, total optimizing coefficients equal *n*_var_ = *N* + 1; fixing values of *C*_1_, *C*_2_ as 1.5; minimum and maximum values of current excitation coefficients, *I*_min⁡_ = 0, *I*_max⁡_ = 1; minimum and maximum values of interelement spacing, *d*_min⁡_ = 0.5*λ*, *d*_max⁡_ = *λ*; initialization of the velocities of all the particle vectors. 

*Step  2*. Generation of initial particle vectors, each vector consisting of current excitation weights and uniform interelement spacing randomly with limits; computation of initial CF values of the total population, *n*_*p*_.

*Step  3*. Computation of population-based minimum CF value and computation of the personal best solution vectors (*pbest*), group best solution vector (*gbest*). 

*Step  4*. Updating the velocities as per (7); updating the particle vectors as per (11), and checking against the limits of current excitation weights coefficients and one number uniform interelement spacing; finally, computation of the updated CF values of the particle vectors and population-based minimum CF value. 

*Step  5*. Updating the *pbest* vectors, *gbest* vector; reuse of the updated particle vectors as initial particle vectors for Step 4. 

*Step  6*. Iteration continues from Step 4 till the maximum iteration cycles or the convergence of minimum CF values; finally, *gbest* is the vector of optimal current excitation weights (*N* number) and uniform interelement spacing (01 number).

### 3.3. Differential Evolution (DE) Algorithm

The crucial idea behind DE algorithm is a scheme for generating trial parameter vectors and adds the weighted difference between two population vectors to a third one. Like any other evolutionary algorithm, DE algorithm aims at evolving a population of *N*_*p*_, *D*-dimensional parameter vectors, so-called individuals, which encode the candidate solutions, that is,

(12) $$\begin{array}{c} {\vec{x}}_{i,g}=\left\{x_{1,i,g},x_{2,i,g},\ldots ,x_{D,i,g}\right\}, \end{array}$$

where *i* = 1, 2, 3,…, *N*_*p*_. The initial population (at *g* = 0) should cover the entire search space as much as possible by uniformly randomizing individuals within the search constrained by the prescribed minimum and maximum parameter bounds:

(13) $$\begin{array}{c} {\vec{x}}_{\min}=\left\{x_{1,\min},\ldots ,x_{D,\min}\right\},\;\;{\vec{x}}_{\max}=\left\{x_{1,\max},\ldots ,x_{D,\max}\right\}. \end{array}$$

For example, the initial value of the *j*th parameter of the *i*th vector is

(14) $$\begin{array}{c} x_{j,i,0}=x_{j,\min}+\text{rand}\left(0,1\right)*\left(x_{j,\max}-x_{j,\min}\right), \end{array}$$

where *j* = 1, 2, 3,…, *D*.

The random number generator, rand(0,1), returns a uniformly distributed random number from within the range [0,1]. After initialization, DE enters a loop of evolutionary operations: mutation, crossover, and selection. 

(a) *Mutation*. Once initialized, DE mutates and recombines the population to produce new population. For each trial vector *x*_*i*,*g*_ at generation *g*, its associated mutant vector ${\vec{v}}_{i,g}=\left\{v_{1,i,g},v_{2,i,g},\ldots ,v_{D,i,g}\right\}$ can be generated via certain mutation strategy. Five most frequently used mutation strategies in the DE codes are listed as follows:

 “DE/rand/1”:
  (15) $$\begin{array}{c} {\vec{v}}_{i,g}={\vec{x}}_{r_{1}^{\prime },g}+F\left({\vec{x}}_{r_{2}^{\prime },g}-{\vec{x}}_{r_{3}^{\prime },g}\right); \end{array}$$
 “DE/best/1”:
  (16) $$\begin{array}{c} {\vec{v}}_{i,g}={\vec{x}}_{\text{best},g}+F\left({\vec{x}}_{r_{1}^{\prime },g}-{\vec{x}}_{r_{2}^{\prime },g}\right); \end{array}$$
 “DE/rand-to-best/1”:
  (17) v→i,g=x→i,g+F(x→best,g−x→i,g)+F(x→r1′,g−x→r2′,g);
 “DE/best/2”:
  (18) v→i,g=x→best,g+F(x→r1′,g−x→r2′,g)+F(x→r3′,g−x→r4′,g);
 “DE/rand/2”:
  (19) v→i,g=x→r1′,g+F(x→r2′,g−x→r3′,g)+F(x→r4′,g−x→r5′,g).

The indices *r*_1_′, *r*_2_′, *r*_3_′, *r*_4_′, *r*_5_′ are mutually exclusive integers randomly chosen from the range [1, *N*_*p*_], and all are different from the base index *i*. These indices are randomly generated once for each mutant vector. The scaling factor *F* is a positive control parameter for scaling the difference vector. *x*_best,*g*_ is the best individual vector with the best fitness value in the population at generation “*g*.” In the present work, (17) has been used.

(b) *Crossover*. To complement the differential mutation search strategy, crossover operation is applied to increase the potential diversity of the population. The mutant vector *v*_*i*,*g*_ exchanges its components with the target vector *x*_*i*,*g*_ to generate a trial vector:

(20) $$\begin{array}{c} {\vec{u}}_{i,g}=\left\{u_{1,i,g},u_{2,i,g},\ldots ,u_{D,i,g}\right\}. \end{array}$$

In the basic version, DE employs the binomial (uniform) crossover defined as

(21) $$\begin{array}{c} u_{j,i,g}=\{\begin{array}{cc} v_{j,i,g} & \text{if}\;\;\left(\text{ran}{\text{d}}_{i,j}\left(0,1\right)\leq C_{r}\;\;\text{or}\;\;j=j_{\text{rand}}\right),\\ x_{j,i,g} & \text{otherwise}, \end{array} \end{array}$$

where *j* = 1, 2,…, *D*.

The crossover rate *C*_*r*_ is user-specified constant within the range (1,0), which controls the fraction of parameter values copied from the mutant vector. *j*_rand_ is a randomly chosen integer in the range [1, *D*]. The binomial crossover operator copies the *j*th parameter of the mutant vector ${\vec{v}}_{i,g}$ to the corresponding element in the trial vector ${\vec{u}}_{i,g}$ if rand_*i*,*j*_(0,1) ≤ *C*_*r*_ or *j* = *j*_rand_. Otherwise, it is copied from the corresponding target vector ${\vec{x}}_{i,g}$. 

(c) *Selection*. To keep the population size constant over subsequent generations, the next step of the algorithm calls for selection to determine whether the target or the trial vector survives to the next generation, that is, at *g* = *g* + 1. The selection operation is described as

(22) $$\begin{array}{c} {\vec{x}}_{i,g+1}=\{\begin{array}{cc} {\vec{u}}_{i,g} & \text{if}\;\;f\left({\vec{u}}_{i,g}\right)\leq f\left({\vec{x}}_{i,g}\right),\\ {\vec{x}}_{i,g} & \text{otherwise}, \end{array} \end{array}$$

where *f*(*x*) is the CF (in this work) to be minimized. So, if the new vector yields an equal or lower value of the objective function, it replaces the corresponding target vector in the next generation; otherwise, the target is retained in the population. Hence, the population either gets better (with respect to the minimization of the objective function) or remains the same in fitness status, but never deteriorates.

The above three steps are repeated generation after generation until some specific termination criteria are satisfied.

#### 3.3.1. Control Parameter Selection of DE

Proper selection of control parameters is very important for the success and performance of an algorithm. The optimal control parameters are problem-specific. Therefore, the set of control parameters that best fit each problem has to be chosen carefully. Values of *F* lower than 0.3 may result in premature convergence, while values greater than 1 tend to slow down the convergence speed. Large populations help maintaining diverse individuals, but also slow down convergence speed. In order to avoid premature convergence, *F* or *N*_*p*_ should be increased or *C*_*r*_ should be decreased. Larger values of *F* result in larger perturbations and better probabilities to escape from local optima, while lower *C*_*r*_ preserves more diversity in the population, thus avoiding local optima.

#### 3.3.2. Algorithmic Description of DE

*Step  1 (generation of initial population)*. Set the generation counter *g* = 0 and randomly initialize *D*-dimensional *N*_*p*_ individuals (parameter vectors/target vectors),

(23) $$\begin{array}{c} {\vec{x}}_{i,g}=\left\{x_{1,i,g},x_{2,i,g},\ldots ,x_{D,i,g}\right\}, \end{array}$$

where *i* = 1,2, 3,…, *N*_*p*_. The initial population (at *g* = 0) should cover the entire search space as much as possible by uniformly randomizing individuals within the search constrained by the prescribed minimum and maximum parameter bounds:

(24) $$\begin{array}{c} {\vec{x}}_{\min}=\left\{x_{1,\min},\ldots ,x_{D,\min}\right\},\;\;{\vec{x}}_{\max}=\left\{x_{1,\max},\ldots ,x_{D,\max}\right\}. \end{array}$$

*Step  2 (mutation).* For *i* = 1 to *N*_*p*_, generate a mutated vector, ${\vec{v}}_{i,g}=\left\{v_{1,i,g},v_{2,i,g},\ldots ,v_{D,i,g}\right\}$ corresponding to the target vector ${\vec{x}}_{i,g}$ via mutation strategy (17). 

*Step  3 (crossover)*. Generation of a trial vector ${\vec{u}}_{i,g}$ for each target vector ${\vec{x}}_{i,g}$ where ${\vec{u}}_{i,g}=\left\{u_{1,i,g},u_{2,i,g},\ldots ,u_{D,i,g}\right\}$, for *i* = 1 to *N*_*p*_; *j*_rand_ = [rand(0,1)∗*D*]; for *j* = 1 to *D*:

(25) $$\begin{array}{c} u_{j,i,g}=\{\begin{array}{cc} v_{j,i,g} & \text{if}\;\;\left(\text{ran}{\text{d}}_{i,j}\left(0,1\right)\leq C_{r}\;\;\text{or}\;\;j=j_{\text{rand}}\right),\\ x_{j,i,g} & \text{otherwise}. \end{array} \end{array}$$

*Step  4 (selection).* For *i* = 1 to *N*_*p*_,

(26) $$\begin{array}{c} {\vec{x}}_{i,g+1}=\{\begin{array}{cc} {\vec{u}}_{i,g} & \text{if}\;\;f\left({\vec{u}}_{i,g}\right)\leq f\left({\vec{x}}_{i,g}\right).\\ {\vec{x}}_{i,g} & \text{otherwise}. \end{array} \end{array}$$

Increment the generation count *g* = *g* + 1.

### 3.4. Collective Animal Behavior (CAB)

CAB is an optimization technique which mimics the collective behaviour of animals [12, 13]. CAB algorithm assumes the existence of a set of operations that resemble the interaction rules that model the collective animal behaviour. In this approach, each solution within the search space represents an animal position. The “fitness value” refers to the animal dominance with respect to the group. The complete process mimics the collective animal behaviour. CAB implements a memory for storing best solutions (animal positions) mimicking the aforementioned biologic process. Such memory is divided into two different elements, one (*M*_*g*_) for maintaining the best locations at each generation and the other (*M*_*h*_) for storing the best historical positions during the complete evolutionary process.

#### 3.4.1. Description of the CAB Algorithm

CAB algorithm [12, 13] is an iterative process that starts by initializing the population randomly (generated random solutions or animal positions). Then, the following four operations are applied until a termination criterion is met (i.e., number of iteration cycles NI):

- keep the position of the best individuals;
- move from or to nearby neighbours (local attraction and repulsion);
- move randomly;
- compete for the space within a determined distance (update the memory).

#### 3.4.2. Initializing the Population

The algorithm begins by initializing a set *A* of   *N*_*p*_ animal positions (*A* = {*a*_1_, *a*_2_,…, *a*_*N*_*p*__}). Each animal position *a*_*i*_ is a *D*-dimensional vector where *D* is equal to the current excitation weights coefficients *N*, and uniform interelement 01 (*n*_var_ = *N* + 1) needs to be optimized. Such values are randomly and uniformly distributed between the prespecified lower initial parameter bound *a*_*j*_^low^ and the upper initial parameter bound *a*_*j*_^high^:

(27) aj,i=ajlow+ rand(0,1)·(ajhigh−ajlow),j=1,2,…,D; i=1,2,…,Np,

*j* and *i* being the parameter and individual indexes, respectively. *a*_*j*,*i*_ is the *j*th parameter of the *i*th individual. All the initial positions *A* are sorted according to the fitness function (dominance) to form a new individual set *X* = {*X*_1_, *X*_2_,…, *X*_*N*_*p*__}, so that the best *B* positions are chosen, which are initially stored in both memories *M*_*g*_ and *M*_*h*_. Thus, both memories share the same information only at this initial stage.

#### 3.4.3. Keep the Position of the Best Individuals

Analogous to the biological metaphor, this behavioural rule, typical from animal groups, is implemented as an evolutionary operation in our approach. In this operation, the first *B* elements ({*a*_1_, *a*_2_,…, *a*_*B*_}), of the new animal position set *A*, are generated. Such positions are computed by the values contained inside the historical memory *M*_*h*_, considering a slight random perturbation around them. This operation is modelled as

(28) $$\begin{array}{c} a_{l}=m_{h}^{l}+v, \end{array}$$

where *l* ∈ {1,2,…, *B*}, while *m*_*h*_^*l*^ represents the *l*-element of the historical memory *M*_*h*_. *v* is a random vector with a small enough length.

#### 3.4.4. Move from or to Nearby Neighbours

From the biological inspiration, animals experiment a random local attraction or repulsion according to an internal motivation. Therefore, new evolutionary operators are implemented that mimic such biological pattern. For this operation, if a uniform random number *r*_*m*_ generated within the range [0,1] is less than a threshold *H*, a determined individual position is attracted/repelled considering the nearest best historical position within the group (i.e., the nearest position in *M*_*h*_); otherwise, it is attracted/repelled to/from the nearest best location within the group for the current generation (i.e., the nearest position in *M*_*g*_). Such operations are modelled as

(29) $$\begin{array}{c} a_{i}=\{\begin{array}{cc} X_{i}\pm r\cdot \left(m_{h}^{\text{nearest}}-X_{i}\right) & \text{with}\;\text{probability}\;H,\\ X_{i}\pm r\cdot \left(m_{g}^{\text{nearest}}-X_{i}\right) & \text{with}\;\text{probability}\;\left(1-H\right), \end{array} \end{array}$$

where *i* ∈ {*B* + 1, *B* + 2,…, *N*_*p*_},  *m*_*h*_^nearest^  and  *m*_*g*_^nearest^ represent the nearest elements of *M*_*h*_ and *M*_*g*_ to *X*_*i*_, respectively, while *r* is a random number within [−1,1]. Therefore, if *r* > 0, the individual position *X*_*i*_ is attracted to the position *m*_*h*_^nearest^ or *m*_*g*_^nearest^; otherwise such movement is considered as repulsion.

#### 3.4.5. Move Randomly

Following the biological model, one animal randomly changes its position under some probability *P*. Such behavioural rule is implemented considering the expression (30) as

(30) $$\begin{array}{c} a_{i}=\{\begin{array}{cc} r & \text{with}\;\text{probability}\;P,\\ X_{i} & \text{with}\;\text{probability}\;\left(1-P\right), \end{array} \end{array}$$

where

(31) $$\begin{array}{c} i\in \left\{B+1,B+2,\ldots ,N_{p}\right\}, \end{array}$$

“*r*” is a random vector defined in the search space. This operator is similar to reinitializing the particle in a random position, as it is done by (27).

#### 3.4.6. Compete for the Space within a Determined Distance (Update the Memory)

Once the operations to keep the position of the best individuals, such as moving from or to nearby neighbours and moving randomly, are applied to all *N*_*p*_ animal positions, generating *N*_*p*_ new positions, it is necessary to update the memory *M*_*h*_. In order to update the memory *M*_*h*_, the concept of dominance is used. Animals that interact within the group maintain a minimum distance among them. Such distance, which is defined as *ρ* depends on how aggressive the animal behaves. Hence, when two animals confront each other inside such distance, the most dominant individual prevails, meanwhile the other withdraws. The historical memory *M*_*h*_ is updated considering the following procedure.

1. The elements of *M*_*h*_ and *M*_*g*_ are merged into *M*_*U*_  (*M*_*U*_ = *M*_*h*_ ∪ *M*_*g*_).
2. Each element *m*_*U*_^*i*^ of the memory *M*_*U*_ is compared pair-wise to the remaining memory elements ({*m*_*U*_^1^, *m*_*U*_^2^,…, *m*_*U*_^2*B*−1^}). If the distance between both elements is less than *ρ*, the element getting a better performance in the fitness function prevails, meanwhile the other is removed.
3. From the resulting elements of *M*_*U*_ (Step 2), the *B* best values are selected to build the new *M*_*h*_.

The computational steps for the CAB algorithm can be summarized as follows.

*Step  1*. Set the population size *N*_*p*_ of vectors (each having *D* number of current excitation weight coefficients *N* and uniform interelement 01 (*n*_var_ = *N* + 1) in *D*-dimensional search space), CAB parameters (*B*, *H*, and *P*), and NI (maximum number of generations).

*Step  2*. Generate randomly the position set *A* = {*a*_1_, *a*_2_,…, *a*_*N*_*p*__} using (27).

*Step  3*. Sort *A* according to the objective function (dominance) to build *X* = {*X*_1_, *X*_2_,…, *X*_*N*_*p*__}.

*Step  4*. Choose the first *B* positions of *X* and store them into the memory *M*_*g*_.

*Step  5*. Update *M*_*h*_ according to (28) (during the first iteration: *M*_*g*_ = *M*_*h*_). 

*Step  6*. Generate the first *B* positions of the new solution set *A* = {*a*_1_, *a*_2_,…, *a*_*B*_}. Such positions correspond to the elements of *M*_*h*_ making a slight random perturbation around them. *a*_*l*_ = *m*_*h*_^*l*^ + *v*; being *v* a random vector of a small enough length. 

*Step  7*. Generate the rest of the *A* elements using the attraction, repulsion, and random movements:

 for *i* = *B* + 1:  *N*_*p*_
 if (*r*_1_ < *P*) then
  (attraction and repulsion movement)
 {if  (*r*_2_ < *H*)  then
 
*a*_*i*_ = *X*_*i*_ ± *r* · (*m*_*h*_^nearest^ − *X*_*i*_)
 else if
 
*a*_*i*_ = *X*_*i*_ ± *r* · (*m*_*g*_^nearest^ − *X*_*i*_)}
 else
 random movement
 {*a*_*i*_ = *r*}
 end
 where *r*_1_, *r*_2_ ∈ rand(0,1) and *r* ∈ [−1,1].

*Step  8*. If maximum number of iteration cycles (NI) is completed, the process is finished; otherwise go back to Step 3. The best value in *M*_*h*_ represents the global solution for current excitation weights coefficients (*N* number) and uniform interelement (01 number).

## 4. Simulation Results

All simulation results were obtained by programming in MATLAB language using MATLAB 7.5 on dual core processor, 2.88 GHz with 2 GB RAM.  Table 1 shows the best chosen parameters for RGA, PSO, DE, and CAB, respectively.

### 4.1. Analysis of Radiation Patterns of Hyperbeam without Optimization

**Figure 4:**
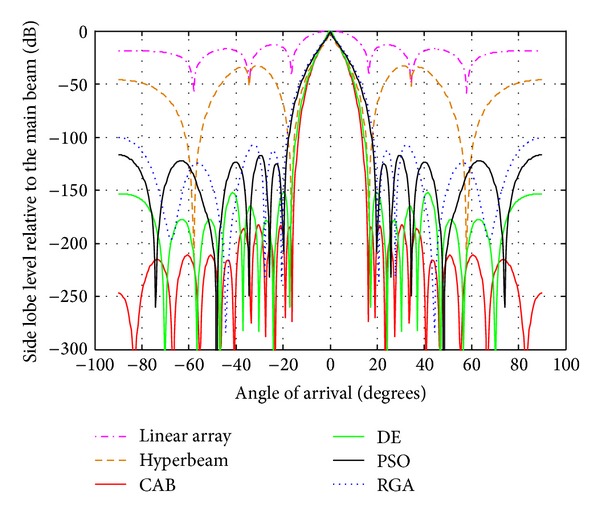
Best array pattern found by CAB for the 10-element array with improved SLL and FNBW at *u* = 0.5.

**Figure 5:**
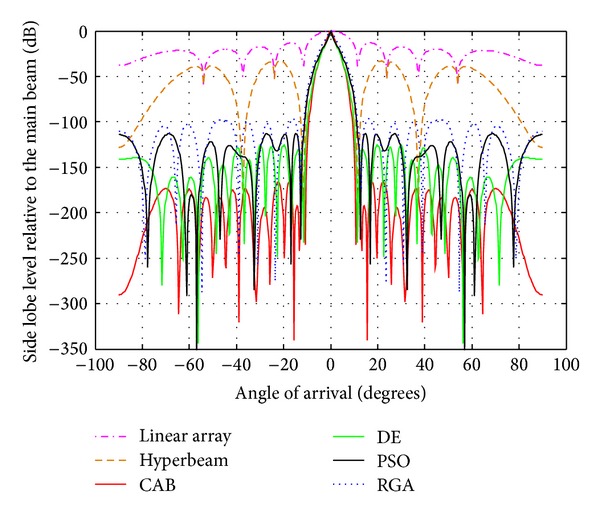
Best array pattern found by CAB for the 14-element array with improved SLL and FNBW at *u* = 0.5.

**Figure 6:**
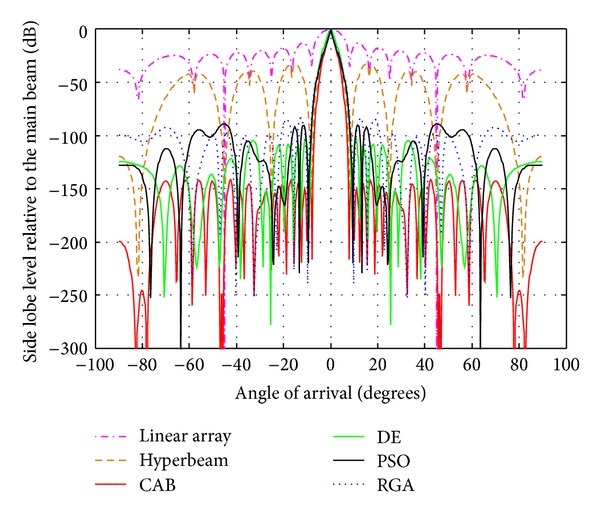
Best array pattern found by CAB for the 20-element array with improved SLL and FNBW at *u* = 0.5.

**Figure 7:**
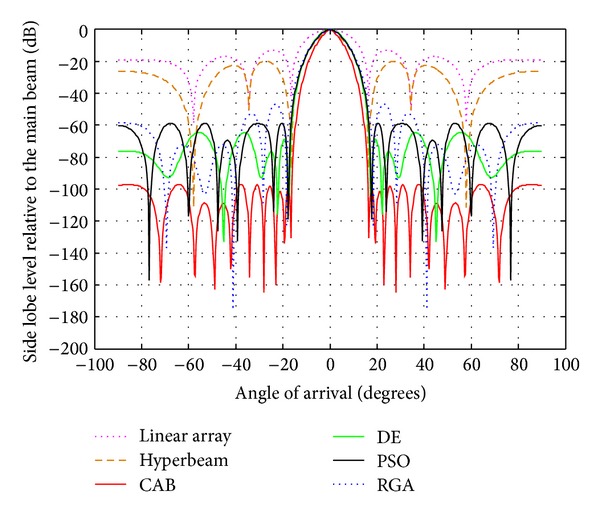
Best array pattern found by CAB for the 10-element array at *u* = 1 with improved SLL.

**Figure 8:**
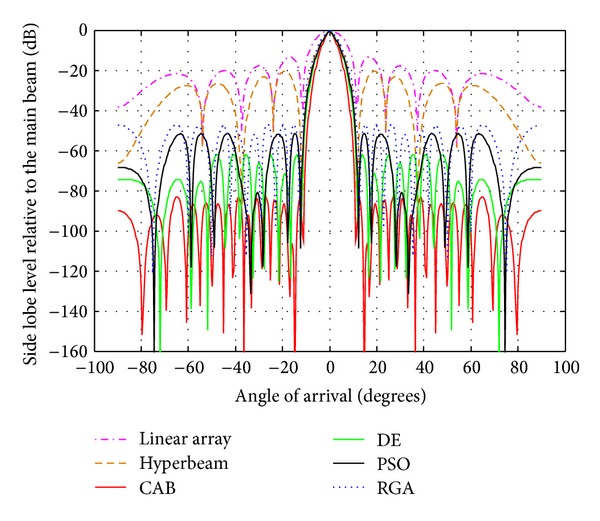
Best array pattern found by CAB for the 14 -element array at *u* = 1 with improved SLL.

**Figure 9:**
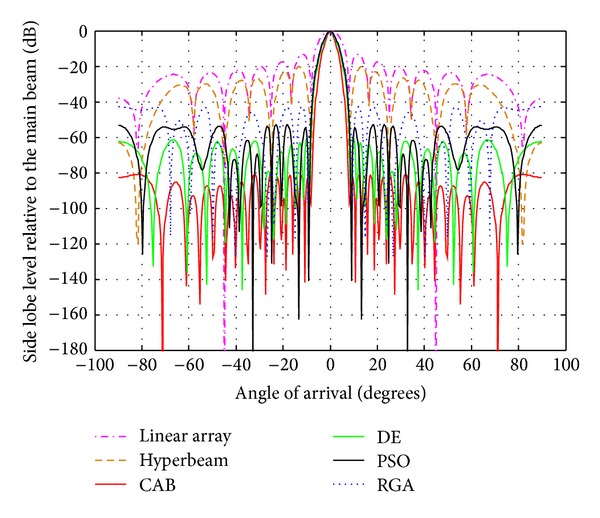
Best array pattern found by CAB for the 20-element array at *u* = 1 with improved SLL.

This section gives the experimental results for various hyper beams of nonoptimized linear antenna array designs. Three linear antenna array designs considered are of 10-, 14-, and 20-element sets, each maintaining uniform interelement spacing. Reduction of SLL can be controlled by varying the hyperbeam exponent value *u*, thereby obtaining different hyperbeam patterns. The results show that the SLL reduction increases as the exponent value *u* decreases. For 10-, 14-, and 20-element linear arrays, with *u* = 1, SLL reductions are −19.91 dB, −20.10 dB, and −20.20 dB, respectively, where as with *u* = 0.5, SLL reduces to −32.78 dB, −33.02 dB, and −33.20 dB, respectively, as shown in Figures 4, 5, 6, 7, 8, and 9 and Table 2. Uniform linear array shows the respective SLL values as −12.97 dB, −13.11 dB, and −13.20 dB. Therefore, in comparison to conventional beamforming, hyperbeam technique yields large reduction of SLL even without any optimization. Main beam width (FNBW) remains unaltered for all cases.

### 4.2. Analysis of Radiation Patterns of Hyper Beams for *u* = 0.5 and 1 with Different Algorithms

This section gives the experimental results for various optimized hyperbeam antenna array designs obtained by RGA, PSO, DE, and CAB techniques. The parameters of the RGA, PSO, DE, and CAB are set after many trial runs. It is found that the best results are obtained for the initial population (*n*_*p*_) of 120 chromosomes and maximum number of generations, *N*_max⁡_ as 100. Each RGA, PSO, DE, CAB individually generates a set of optimized, nonuniform current excitation weights and optimal uniform interelement spacing for same three sets of linear antenna arrays. Tables 3 and 4 show SLL, FNBW, optimal current excitation weights with hyperbeam exponent value *u* = 0.5, and *u* = 1, respectively, for optimally excited hyperbeam linear antenna array with optimized uniform interelement spacing (*d* ∈ [*λ*/2, *λ*]) using RGA, PSO, DE, and CAB. Figures 4–9 depict the radiation patterns of linear antenna arrays with the exponent values *u* = 0.5 and *u* = 1 for sets of 10, 14, and 20 number of elements, respectively, with optimized nonuniform excitations and optimized fixed interelement spacing, as obtained by the techniques. Figures clearly show improvement of SLL and FNBW by optimization of hyper beam.

The following observations are made from Table 3 and Figures 4, 5, and 6, in which the exponent value *u* = 0.5. The algorithms yield SLL values of −100.6 dB (RGA), −117.2 dB (PSO), −151.9 dB (DE), and −182.8 dB (CAB) for the 10-element array, then, −96.21 dB (RGA), −113 dB (PSO), −125.8 dB (DE), and −166 dB (CAB) for the 14-element array, and finally, −83.69 dB (RGA), −88.71 dB (PSO), −101.9 dB (DE), and −142.1 dB (CAB) for the 20-element array of respective optimized hyperbeam patterns against SLL of −32.78 dB, −33.02 dB, and −33.20 dB of respective nonoptimized hyperbeam patterns. Regarding FNBW values for the same respective arrays, the algorithms yield 41.04 degrees (RGA), 39.60 degrees (PSO), 34.56 degrees (DE), and 32.4 degrees (CAB), then, 25.92 degrees (RGA), 25.20 degrees (PSO), 23.04 degrees (DE), and 20.16 degrees (CAB), and finally, 19.44 degrees (RGA), 18.72 degrees (PSO), 18 degrees (DE), and 15.84 degrees (CAB) of respective optimized hyperbeam patterns against FNBW of 33.12 degrees, 23.04 degrees, and 16.56 degrees of respective nonoptimized hyperbeam patterns. Thus, Figures as well as Tables clearly show much improvement of both SLL and FNBW by CAB-based optimization, as compared to the other algorithms.

From Table 4 and Figures 7, 8, and 9, in which the exponent value *u* = 1.0, the same nature of observations can be made with regard to SLL and FNBW values for the algorithms. In this case, also, CAB proves its superiority in yielding better SLL and FNBW as compared to the other algorithms. CAB efficiently computes *N* number of near global optimal current excitation weights and one number optimal uniform interelement separation for each hyperbeam linear antenna array to have maximum SLL reduction and much improved FNBW.

## 5. Convergence Profiles of RGA, PSO, DE, and CAB

**Figure 10:**
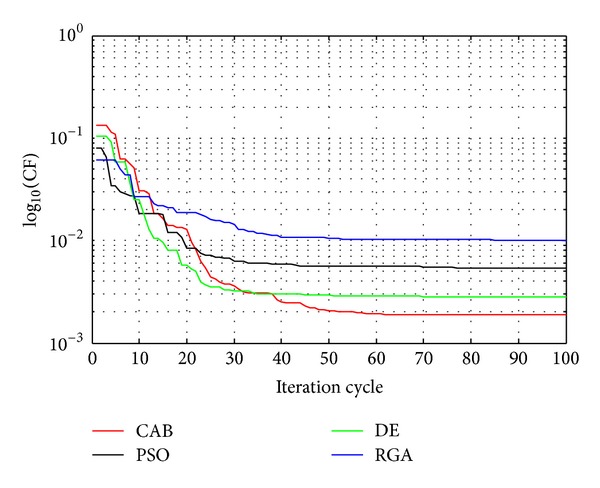
Convergence profile of CAB in case of 10-element linear antenna array at *u* = 0.5.

**Figure 11:**
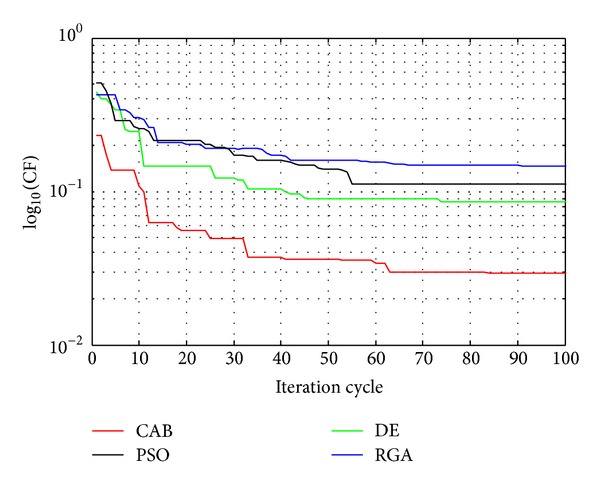
Convergence profile of CAB in case of 10-element linear antenna array at *u* = 1.

The algorithms can be compared in terms of the cost function (CF) values, Figures 10 and 11 show the convergences of  log⁡_10_(CF) values obtained for 10-element array sets, for *u* = 0.5 and 1, respectively, as RGA, PSO, DE, and CAB are employed, respectively. CAB converges to the least minimum CF as compared to RGA, PSO, and DE which yield suboptimal higher values of CF. CAB thus yields the near-global optimal current excitation weights and optimal inter element spacing of hyperbeam of linear antenna arrays.  Table 5 shows the execution times of RGA, PSO, DE, and CAB. From the same table, it is clear that execution times of CAB are lesser than those of RGA and DE but not those of PSO. With a view to the above fact, it may be inferred that the performance of the proposed CAB algorithm is the best among the algorithms for solving the optimization problem of hyper beamforming design.

## 6. Conclusions

In this paper, a novel algorithm based on collective animal behaviour (CAB) is used for finding the best optimal nonuniform excitation weights, *I*_*n*_ (0 < *I*_*n*_ ≤ 1) and optimal uniform interelement spacing, *d* (*λ*/2 ≤ *d* < *λ*) for hyper beamforming of linear antenna arrays. Three broad cases of arrays are considered in the study. The first two cases are (i) conventional uniformly excited (*I*_*n*_ = 1) linear antenna arrays with interelement spacing, *d* = *λ*/2 and (ii) nonoptimized uniformly excited (*I*_*n*_ = 1) hyper beamforming of linear antenna arrays with interelement spacing, *d* = *λ*/2. The last one is of actual concern, which is hyper beamforming of linear antenna arrays with optimized interelement spacing (*λ*/2 ≤ *d* < *λ*) along with optimized nonuniform excitations (0 < *I*_*n*_ ≤ 1). The optimization algorithms considered are RGA, PSO, DE, and CAB. Extensive experimental results reveal that the other algorithms RGA, PSO, and DE are entrapped to suboptimal designs. Whereas the collective animal behaviour (CAB) yields optimal designs, offering in the highest reduction in sidelobe level (SLL) and much more improved first null beam width (FNBW) as compared to the other two cases for any hyperbeam exponent parameter.

## Figures and Tables

**Table 1:** RGA, PSO, DE, and CAB parameters.

Parameters	RGA	PSO	DE	CAB
Population size	120	120	120	120
Iteration cycle	100	100	100	100
Crossover rate	0.8	—	—	—
Crossover	Two point cross over	—	—	—
Mutation rate	0.05	—	—	—
Mutation	Gaussian mutation	—	—	—
Selection, probability	Roulette wheel, 1/3	—	—	—
*C*_1_, *C*_2_	—	1.5, 1.5	—	—
*v*_*i*_^min⁡^, *v*_*i*_^max⁡^	—	0.01, 1.0	—	—
*w*_max⁡_, *w*_min⁡_	—	1.0, 0.4	—	—
*C*_*r*_	—	—	0.3	—
*F*	—	—	0.5	—
*B*, *P*, *H*				20, 0.5, 0.5

**Table 2:** Initial values of SLL and FNBW for uniform linear array having uniform excitation (*I*_*n*_ = 1) and λ/2 inter-element spacing.

*N*	SLL (dB) for uniform linear array with (*I*_*n*_ = 1) and λ = 0.5	SLL of hyper beam nonoptimized (dB) at *u* = 0.5, (*I*_*n*_ = 1) and λ = 0.5	SLL of hyper beam nonoptimized (dB) at *u* = 1, (*I*_*n*_ = 1) and λ = 0.5	FNBW (deg) for uniform linear array with (*I*_*n*_ = 1) and λ = 0.5	FNBW of hyper beam nonoptimized (deg) at *u* = 0.5, (*I*_*n*_ = 1) and λ = 0.5	FNBW of hyper beam nonoptimized (deg) at *u* = 1, (*I*_*n*_ = 1) and λ = 0.5
10	−12.97	−32.78	−19.91	33.12	33.12	33.12
14	−13.11	−33.02	−20.10	23.04	23.04	23.04
20	−13.20	−33.20	−20.20	16.56	16.56	16.56

**Table 3:** SLL, FNBW, optimal current excitation weights, and optimal inter-element spacing for hyper beam pattern of linear array with hyper beam exponent (*u* = 0.5), obtained by RGA, PSO, DE, and CAB for different sets of arrays.

*N*	Algorithms	Optimized current excitation weights and [*I*_1_, *I*_2_, *I*_3_, *I*_4_,…, *I*_*N*_]	Optimal inter-element spacing in (λ)	SLL of hyper beam with optimization (dB)	FNBW of hyper beam with optimization (deg)
	RGA	0.2844 0.5240 0.8813 0.9032 0.4231 0.8425 0.4564 0.6402 0.3414 0.3853	0.5441	−100.6	41.04
10	PSO	0.2398 0.6414 0.9123 0.9722 0.4312 0.9502 0.4327 0.6582 0.3571 0.3982	0.5717	−117.2	39.60
	DE	0.1029 0.3802 0.6258 0.9394 0.7907 1.0000 0.5211 0.5538 0.2118 0.2156	0.8470	−151.9	34.56
	CAB	0.2359 0.3120 0.4490 0.4396 0.9983 0.7164 0.9233 0.7213 0.3960 0.0846	0.8594	−182.8	32.4

	RGA	0.3631 0.2555 0.4905 0.0043 0.6114 0.5778 0.8634 0.5042 0.5782 0.5913 0.7502 0.5545 0.2878 0.3431	0.5878	−96.21	25.92
14	PSO	0.2319 0.1857 0.6027 0.5089 0.7906 0.4163 0.6275 0.7212 0.9097 0.2907 0.2525 0.2755 0.5506 0.3615	0.6036	−113	25.20
	DE	0.2297 0.3701 0.3080 0.2229 0.6599 0.9495 0.6941 0.8597 0.4157 0.7559 0.7305 0.2389 0.3759 0.0982	0.7949	−125.8	23.04
	CAB	0.0510 0.2113 0.3799 0.8250 0.9015 0.8437 0.9090 0.9410 0.8698 0.5521 0.3112 0.4662 0.3493 0.1664	0.9245	−166	20.16

	RGA	0.2505 0.3933 0.4881 0.4829 0.3027 0.6697 0.3436 0.9551 0.5974 0.8952 0.5252 0.9773 0.4056 0.6612 1.0000 0.1577 0.8144 0.3284 0 0.5558	0.5361	−83.69	19.44
20	PSO	0.1675 0.2453 0.2113 0.5168 0.6011 0.5661 0.7962 0.2148 0.8279 0.2476 0.9888 0.3429 0.8064 0.1836 0.2281 0.1792 0.4317 0.6579 0.2244 0.3467	0.5353	−88.71	18.72
	DE	0.1567 0.1345 0.5561 0.4817 0.9529 0.7651 0.9420 0.7511 0.6736 0.5927 0.9889 0.8862 0.4313 0.4025 0.2891 0.3316 0.4286 0.4649 0.4306 0.3195	0.5852	−101.9	18
	CAB	0.1411 0.4085 0.4124 0.4649 0.4193 0.4515 0.5774 0.7569 0.9998 0.9478 0.9593 0.8373 0.9730 0.9216 0.8734 0.6891 0.4313 0.2586 0.0089 0.0987	0.8283	−142.1	15.84

**Table 4:** SLL, FNBW, optimal current excitation weights, and optimal inter-element spacing for hyper beam pattern of linear array with hyper beam exponent (*u* = 1), obtained by RGA, PSO, DE, and CAB for different sets of arrays.

*N*	Algorithms	Optimized current excitation weights and [*I*_1_, *I*_2_, *I*_3_, *I*_4_,…, *I*_*N*_]	Optimal inter-element spacing In (λ)	SLL of hyper beam with optimization (dB)	FNBW of hyper beam with optimization (deg)
	RGA	0.1339 0.1010 0.4353 0.3657 0.6166 0.5295 0.6264 0.4194 0.3935 0.2296	0.6503	−46.76	36.72
10	PSO	0.3889 0.4254 0.2096 0.7456 0.7961 0.4382 0.3525 0.5002 0.1764 0.1603	0.6436	−58.88	35.28
	DE	0.2057 0.4820 0.9658 0.9686 1.0000 0.9881 0.5582 0 0 0.0086	0.9974	−64.57	34.56
	CAB	0.1692 0.3915 0.4476 0.6693 0.9456 0.9860 0.9550 0.6883 0.2226 0.0156	0.9358	−96.96	33.12

	RGA	0 0.4146 0.6005 0.7859 0.7903 0.7755 0.4159 0.9358 0.2159 0.3125 0.4533 0 0.6501 0.1934	0.5824	−46.76	25.20
14	PSO	0.1011 0.2588 0.3020 0.5343 0.6365 0.6937 0.5245 0.8198 0.3813 0.4761 0.3815 0.4803 0.1301 0.3374	0.6698	−51.4	24.48
	DE	0.1822 0.4092 0.3052 0.3611 0.5660 0.8365 0.6771 0.7047 0.4664 0.6376 0.5091 0.3593 0.0822 0.1951	0.7436	−61.71	23.76
	CAB	0.2313 0.2924 0.4580 0.3613 0.9441 0.9963 0.9818 0.9922 0.8262 0.6192 0.8685 0.3357 0.1659 0.1938	0.9253	−82.6	22.32

	RGA	0.2739 0.0772 0.4652 0.3369 0.4341 0.6162 0.5613 0.8008 0.4211 0.7082 0.6840 0.8283 0.3579 0.4822 0.3872 0.7091 0.3145 0.3415 0.1838 0.4675	0.5587	−42.85	18.72
20	PSO	0.5918 0.0903 0.4110 0.0131 0.6447 0.1519 0.7800 0 0.8548 0.8593 0.6530 0.7593 0.9763 0.9991 0.7571 0.8972 0.5175 0.7424 0.2818 0.2433	0.5961	−52.97	18
	DE	0.0938 0.1489 0.2668 0.5640 0.6043 0.8496 0.8847 0.7082 0.6143 0.7392 0.8163 0.8767 0.7024 0.3612 0.2068 0.3691 0.3646 0.5144 0.3815 0.1665	0.6638	−61.19	17.28
	CAB	0 0.0048 0.1938 0.4010 0.6162 0.7142 0.9277 0.9825 0.8613 0.8899 0.9679 0.9160 0.6405 0.5015 0.5205 0.3977 0.3407 0.3297 0.3303 0.1310	0.9297	−80.55	16.56

**Table 5:** Comparison of execution times for different algorithms for different sets of elements.

No. of elements	Execution times for different algorithms			
	RGA (sec)	DE (sec)	PSO (sec)	CAB (sec)
10	384.840	311.3459	244.2920	286.0470
14	408.2940	376.4710	289.0360	310.2220
20	538.4420	483.5892	320.0520	358.5028
